# The Nutritional Facts of Bamboo Shoots and Their Usage as Important Traditional Foods of Northeast India

**DOI:** 10.1155/2014/679073

**Published:** 2014-07-20

**Authors:** P. Nongdam, Leimapokpam Tikendra

**Affiliations:** Department of Biotechnology, Manipur University, Canchipur, Imphal, Manipur 795003, India

## Abstract

Bamboo shoots are considered as one of the useful health foods because of their rich contents of proteins, carbohydrates, vitamins, fibres, and minerals and very low fat. Though bamboo shoots provide lots of health benefits, their consumption is confined mostly to Southeast Asian and East Asian countries. The acceptability of bamboo shoots as popular vegetable crop is very less due to their high pungent smell and bitter acidic taste. The use of bamboo as food in India is mainly restricted to Northeastern part of the country where they form an indispensable part of several traditional speciality dishes. The different ethnic communities take fresh or fermented bamboo shoot as one of most preferred traditional food items. Some of the important bamboo based traditional foods are *ushoi, soibum, rep, mesu, eup, ekhung, hirring*, and so forth. Bamboo shoots should be properly processed before they are consumed as freshly harvested shoots have high content of toxic cyanogenic glycosides which may pose serious health problems. The prospect of bamboo shoot industry in Northeast India is bright due to its rich genetic resources of bamboos. However, habitat destruction and extensive use of bamboos for food, handicraft, and construction purposes have resulted in severe depletion of natural bamboo resources. This review stresses upon the high nutritive values and health benefits of bamboo shoots and their usage as important traditional foods in Northeast India. The bamboo market potential of the region and use of *in vitro* plant micropropagation methods as effective means of bamboo conservation are also emphasized in this paper.

## 1. Introduction

Bamboos belonging to family Poaceae are considered as one of the most versatile multiutility forest tree grasses. Though distribution of bamboos is worldwide with over 1250 species, their presence is predominantly found in Southeast Asia [1, 2]. They are known to have more than 1500 uses and are considered as one of the most economically important plants in the world [3]. The applicability of bamboos is highly diverse as they are employed immensely in paper, handicraft industry, house construction, and making furniture, water pipes, storage vessels and other important household items [4]. People from different countries address bamboos in different names because of their highly multipurpose properties. The Chinese called bamboos as “Friends of the people,” Vietnamese as “My brother,” and Indians as “Green Gold.” Bamboos in addition to their multiple applications have another important usage in utilizing their juvenile shoots as popular food items. The presence of high content of protein, amino acids, minerals, fibre, carbohydrates, and low fat makes the bamboo shoot one of the widely acclaimed nutrient rich food items. Also the presence of phytosterols in young shoots provides youthful feeling, athletic energy, and longevity to regular consumers. Bamboos shoots are popular in Asiatic countries and form a major component of their traditional cuisines [5]. The people of Northeast India with their mongoloid features are endowed with rich bamboo culture and the plants are inseparable part of several diverse traditions and religious beliefs of many ethnic people residing at both hilly and plain areas. Consumption of bamboo shoots as food in India is mainly confined to the Northeast states where they are taken either fresh at the time of harvesting season or dried, fermented or pickled forms during offseason [6]. The bamboo shoots are the integral constituents of many of the popular traditional cuisines. The present review explores the nutritive values and health benefits of bamboo shoots and the necessity of proper processing methods to generate nontoxic consumable bamboo products. The use of bamboo shoots as traditional foods of Northeast India and the potential and prospect of bamboo shoot industry in the region along with the importance of effective bamboo conservation through micropropagation are also highlighted.

## 2. Nutrient Composition of Bamboo Shoots

Bamboo shoots have immense potential of being used as important health food as they contain high proteins, amino acids, carbohydrates, many important minerals, and vitamins [12]. Freshly collected bamboo shoots have good amount of thiamine, niacin, vitamin A, vitamin B6, and vitamin E [13, 14]. Also the bamboo shoot based diets are rich source of dietary fibres and phytosterols and less cholesterol contents which make them one of the popular natural health foods.  Table 1 shows the comparison of nutritive values of shoots of edible bamboos with some of the popularly consumed vegetables in different parts of the world. The nutrient compositions of shoots of different edible bamboo species have been analysed by several workers [6, 13, 15–18]. The nutrient contents of some important edible bamboo species is given in Table 2. Bamboo shoots contain generally tyrosine, arginine histidine, and leucine as amino acids. The presence of tyrosine facilitates biochemical metabolism of our body as it is a major constituent of adrenals which are precursors for adrenaline, necessary for active body metabolic activities. It also plays important role in function of thyroid and pituitary glands which are involved in producing and regulating hormones in human body. Presence of high fibre and phytosterols in bamboo shoot reduces fat and cholesterol levels of blood making them one of the most sought after health foods among patients with life style related disorders. The dietary fibre possesses number of health benefits as it controls blood pressure, hypertension, and obesity and also protects our body from coronary diseases and potential carcinogens [19, 20]. The survey conducted by Kalita and Dutta [21] showed that some ethnic tribes of Northeast India used bamboo shoots to control high blood pressure and cardiovascular ailments. The high dietary fibres and low fat in bamboo shoot help in reducing the thickening of arteries maintaining the blood pressure. Park and John [22] conducted a study to show that diet containing bamboo shoots had reducing effect on serum content of total cholesterol and low density lipoprotein. There was increase in the frequency of bowel movement and faecal volume indicating its role in cholesterol lowering and diabetes prevention in individuals provided with bamboo diets. There are instances of using bamboo shoots by Karbi Anglong tribes of India to control early stage of cancer [21]. The anticancer property of bamboo shoots might be attributed to the presence of lignans and phytosterols. The production of carcinogens, growth of cancer cells, cell invasion, and metastasis are inhibited by phytosterol [23]. Regular intake of bamboo shoots reduces reproductive health related problems in female. Bamboo shoots are used by local tribes belonging to Bodo, Thadau, Mosang, and Tiwa for treatment of irregular menstrual cycle, heavy bleeding after delivery, infertility problems, reducing labour pain, and also for inducing puberty in young female. Though scientifically not proven some tribes believe bamboo shoot causes abortion in pregnant women. They are often advised not to consume bamboo shoot during the first trimester of pregnancy.

### 2.1. Amino Acids

Bamboo shoots have rich amount of amino acids. Out of 17 amino acids reported in bamboo shoots, 8 amino acids were essential for human body [24]. Nirmala et al. [6] studied amino acid content of freshly harvested, fermented, and canned shoots of* Dendrocalamus giganteus*. There was decreased amount in amino acid in fermented (2.005 g/100 g fresh weight) and canned shoots (1.980 g/100 g fresh weight) as compared to freshly collected juvenile shoots (3.863 g/100 g fresh weight). Giri and Janmejay [25] reported reduction of individual amino acid content in 300-day-old bamboo shoots of* Bambusa tulda*. Kozukuen et al. [26] observed the presence of tyrosine as the most abundantly free amino acids in young shoots of* Phyllostachys pubescens*. The amount of amino acids in bamboo shoots of five species of* Bambusa *and another four species of* Dendrocalamus *was found to be highly variable [17]. There was significant decrease in free amino acid in 10-day-old shoots of* Bambusa bamboos*,* Bambusa tulda, Dendrocalamus asper, Dendrocalamus giganteus,* and* Dendrocalamus hamiltonii *as compared to freshly harvested shoots [27]. Zhang et al. [28] detected 12 free amino acids in shoots of* Phyllostachys praecox*. The six amino acids, namely, aspartate, glutamine, glycine, alanine, tyrosine, and histidine were found to be nonessential and remaining 6 amino acids, namely, valine, methionine, isoleucine, leucine, and lysine were essential amino acids. There was substantial decrease in total free essential amino acid (TFEAA) and total free amino acid (TFAA) in boiled bamboo shoots by about 38.85% and 38.35%, respectively. This might be due to loss of amino acids to fluid when the samples were heat treated. Xu et al. [29] evaluated the amino acids components of 9 bamboo species from South Ankyi Province of China using regular methods. The essential amino acids represented 12–49% of total amino acid content of bamboo shoots under study. Chen et al. [30] examined the amino acid quantity of shoots of* Dendrocalamus latiflorus* in different salt concentrations during pickling process. The amino acid in pickled bamboo shoots with 8% salt concentration was highly reduced from 16.35 g/100 g fresh weight in fresh form to 6.898 g/100 g fresh weight in pickled shoot.

### 2.2. Proteins

Bamboo shoots are good source of protein with protein content ranging from 1.49 g/100 g to 4.04 g/100 g fresh weight in fresh bamboo shoots [7, 31]. Sharma et al. [17] found variation in amount of protein in 9 species of bamboos studied. Pandey and Ojha [32] observed gradual decrease in protein content in shoots of* Bambusa bamboos* when shoots were subjected to heat treatment in increase salt concentration. The level of protein was least reported in shoots boiled for 25 minutes in 10% NaCl solution. Nirmala et al. [6] detected the lowest amount of protein in canned shoots (1.980 g/100 g fresh weight) as compared to fermented (2.005 g/100 g fresh weight), 10-day-old emerged shoots (2.230 g/100 g fresh weight) and freshly harvested shoots (3.863 g/100 g fresh weight) of* Dendrocalamus giganteus*. However Devi and Singh [33] found enhancement of protein content in fermented shoots from 3.1% to 7.1% and 8.1% on the 3rd and 5th day of fermentation process, respectively. Zhang et al. [28] reported decrease in level of protein in bamboo shoots in* Dendrocalamus asper* and* Dendrocalamus strictus* with time. The protein content of 8-day-old bamboo shoot of* Dendrocalamus asper* (1.21 g/100 g fresh weight) and* Dendrocalamus strictus* (1.91 g/100 g fresh weight) reduced significantly in another 10 days as observed in 18-day-old shoots of* Dendrocalamus asper *(0.86 g/100 g fresh weight). Another observation made by Nirmala et al. [27] indicated the decrease in protein level in 10-day-old bamboo shoots when compared to freshly harvested shoots of* Bambusa bamboos, Bambusa tulda, Dendrocalamus asper, Dendrocalamus giganteus,* and* Dendrocalamus hamiltonii*.

### 2.3. Minerals

Bamboo shoots are endowed with rich quantities of useful minerals such as potassium, phosphorus, sodium, calcium, magnesium, and iron. Minerals are required for the proper functioning of many useful metabolic activities of our body. Bhargava et al. [10] identified the highest content of potassium (1400 mg/100 g fresh weight) in* Bambusa arundinaria* while the least amount (20 mg/100 g fresh weight) was detected in* Melocanna baccifera*. Bhatt et al. [8] reported low amount of potassium (20 mg/100 g fresh weight) in* Dendrocalamus longispathus*,* Dendrocalamus hookeri, Dendrocalamus sikkimensis,* and* Bambusa tulda*. Nirmala et al. [27] observed very slight reduction in potassium content in 10-day-old shoots of* Bambusa bamboos, Bambusa tulda, Dendrocalamus asper,* and* Dendrocalamus giganteus* except for* Dendrocalamus hamiltonii* which had potassium amount of fresh shoot (416 mg/100 g fresh weight) reduced to almost half (210 mg/100 g fresh weight). Feleke [34] reported high amount of potassium in bamboo shoots of* O. abyssinica* collected from Assosa (6.63%), Dhidhessa (7.02%), and Pawe (7.15%) regions of Ethiopia. The level of potassium in shoots of* O. Abyssinica *was higher than the popular vegetables like* Amaranthus spinosus, Hibiscus *species, and* Solanum macrocarpon*. The daily recommended dose of potassium is 2.0 to 5.5 g/d [35] and it confers protection to human heart by maintaining normal BP and stable heartbeat of an individual. The iron requirement by women at pregnancy and during the nursing of child is very high [36]. Regular consumption of iron rich bamboo shoots will provide the necessary iron requirement of the individual. Pandey and Ojha [32] examined changes in mineral content of shoots of* Bambusa bamboos, Bambusa tulda, Dendrocalamus asper,* and* Dendrocalamus strictus* by subjecting to differential treatment of salt concentration and boiling at different time durations. The minerals, namely, potassium, sodium, phosphorus, calcium, and magnesium present in the fresh bamboo shoot did not show significant change after treatments. However level of sodium enhanced prominently from 0.07 mg/100 g fresh weight in fresh shoot to 1.15 mg/100 g fresh weight in shoots boiled in 10% NaCl for 25 minutes. There is slight variation in presence of traces elements such as cadmium, cobalt, manganese, nickel, and selenium in freshly harvested shoots, fermented and nonsalted canned shoots in* Dendrocalamus giganteus*. But reduction in zinc content was observed in canned and fermented shoots as compared to fresh shoots [6].

### 2.4. Carbohydrate

The level of carbohydrates present in bamboo shoots is reported to be high and its content in edible shoots of* Bambusa nutans, Bambusa vulgaris, Dendrocalamus strictus,* and* Dendrocalamus asper* was found at 3.3%, 3.4%, 0.6%, and 2.9%, respectively [11]. There was significant increase in the amount of carbohydrate in bamboo shoots after subjecting them to boiling process. The rise in carbohydrate may be due to hydrolysis of complex polysaccharides into single monosaccharide sugar units. The carbohydrate content decreased when bamboo shoots from* Bambusa bamboos, Bambusa tulda, Dendrocalamus asper,* and* Bambusa strictus *were boiled in solution with different salt concentrations [32]. The presence of salt in solution might have played a role in reducing the carbohydrate level in shoot by enhancing the hydrolysis of carbohydrate during boiling. Pandey and Ojha [37] also reported a rise in carbohydrate level with increase in age of bamboo shoots. The carbohydrate content in two-day-old shoot (1.42 g/100 g fresh weight) increased to 2.46 g/100 g fresh weight after 16 days. However, Nirmala et al. [27] reported reduction of carbohydrate with increase in age of bamboo as 10-day-old shoot (2.30 g/100 g fresh weight) had lesser carbohydrate as compared to freshly harvested shoots (5.42 g/100 g fresh weight). Carbohydrate in fermented shoot (1.504 g/100 g fresh weight) was decreased when compared to carbohydrate content of fresh shoots of* Dendrocalamus giganteus *(5.103 g/100 g fresh weight) [6]. Similar observation of carbohydrate reduction was reported by Devi and Singh [33] in* soidon, *produced from fermented shoots of* Phyllostachys humilis*.

### 2.5. Fat

Bamboos shoots are known to have very less amount of fats and its low content makes them an ideal candidate for providing healthy nutrition to people with diabetic and cardio thoracic diseases. Kumbhare and Bhargava [11] reported very low amount of fat (0.1 g/100 g fresh weight) in shoots of* Dendrocalamus strictus*. Nirmala et al. [6] also revealed lowest fat content in nonsalted canned shoots (0.250 g/100 g fresh weight) as compared to fermented (0.315 g/100 g fresh weight) and freshly harvested shoots (0.387 g/100 g fresh weight) of* Dendrocalamus giganteus*. The level of fat was significantly increased almost 3- to 4-fold in older shoots of 10 days as compared to juvenile shoots of* Bambusa bamboos, Bambusa tulda, Dendrocalamus asper, Dendrocalamus giganteus,* and* Dendrocalamus hamiltonii* [27]. Bhatt et al. [8] reported maximum fat level (1.00 g/100 g fresh weight) in* Bambusa nutans* while the least was observed in* Bambusa polymorpha, Bambusa vulgaris, *and* Dendrocalamus strictus*. Kozukue and Kozukue [38] detected presence of fatty acids like palmitic, linoleic, and linolenic acid in bamboo shoots. The level of fat was reduced in boiled and steamed bamboo shoots of* Phyllostachys praecox *except for stirred fried bamboo shoots which had total gain of fat from 0.21 g/100 g fresh weight in raw shoot to 1.32 g/100 g fresh weight in stirred food [28].

### 2.6. Fibre

The dietary fibres possess number of health benefits as they control blood pressure, hypertension, and obesity and also protect body from coronary diseases and potential carcinogens [19]. The consumption of fibre-rich diets helps in reducing the unwanted bad cholesterols (low density lipoprotein and very low density lipoprotein) in the blood, lowering insulin demand, keeping the digestive track healthy, and improving laxative property [39–41]. The intake bamboo shoots on regular basis improves the lipid profile and bowel movement in young healthy women [8, 42]. Bhatt et al. [8] reported maximum presence of crude fibre (35.5%) in* Melocanna baccifera,* while Sharma et al. [17] observed variation in total fibre, cellulose, hemicellulose, and lignin content in bamboo shoots of* Bambusa arundinaria*,* Bambusa polymorpha*,* Bambusa tulda*,* Dendrocalamus vulgaris*,* Dendrocalamus calostachyus, Dendrocalamus giganteus*,* Dendrocalamus membranaceus,* and* Dendrocalamus strictus*. The amount of dietary fibre increased by around 2fold in 10-day-old shoots as compared to fresh shoots of* Bambusa bamboos*,* Bambusa tulda, Dendrocalamus asper*,* Dendrocalamus giganteus,* and* Dendrocalamus hamiltonii* [27]. The fibre content in bamboo shoot after boiling was not decreased significantly [11]. However dietary fibre components such as NDF, ADF, lignin, hemicellulose, and cellulose increased in fermented shoots of* Dendrocalamus giganteus *when compared to freshly harvested shoots [7]. The presence of high fibre in bamboo shoots helps in reducing the fat and cholesterol level of blood thereby making them one of popular health foods among individual with modern lifestyle disease. Pandey and Ojha [37] reported the increase in dietary fibre in the shoots of* Dendrocalamus asper, Dendrocalamus strictus,* and* Bambusa tulda* with increase in age of bamboo shoots. The comparative analysis of nutrient composition of fresh, boiled, and fermented bamboo shoots of some edible bamboo species is described in Table 3.

### 2.7. Phytosterol

Phytosterols with its structure similar to cholesterol are extensively found in plants and their presence in fresh or fermented bamboo shoots is very prominent [43, 44]. The importance of phytosterol in maintaining quality life is well documented by several workers [45–47]. Lu et al. [48] evaluated phytosterol content in bamboo shoots of* Pleioblastus amarus, Pleioblastus pubescens, Dendrocalamus latiflorus,* and* Pleioblastus praecox* using ultraperformance liquid chromatography. They detected presence of higher level of *β*-sitosterol as compared to other sterols like campesterol and stigmasterol. Phytosterol-rich diets help in reduction of colon, breast, and prostate cancer [49–51]. The progression of tumour growth is inhibited by interfering the cell cycle, apoptosis, and tumour metastasis. The cholesterol level in blood is reduced as presence of phytosterol inhibits the absorption of dietary cholesterol and cholesterol esterification in intestinal mucosa. Lachance and He [52] identified *β*-sitosterol, campesterol, and stigmasterol as the major sterols present in bamboo shoots. Sarangthem and Singh [53] reported sitosterol as the most abundantly available phytosterol in bamboo shoots. A good amount of phytosterol which can be extracted from freshly harvested bamboo shoots may be effectively employed for the production of steroidal drugs. This will ease the pressure on* Dioscorea* and* Solanum* species as primary natural source of steroidal drugs.

### 2.8. Phenols

Phenolic compounds present in plants are important bioactive compounds as they exhibit strong natural antioxidative and anti-inflammatory properties and sometimes antimicrobial activities as well [54–57]. Bamboo leaves possess antioxidant capacity because of high presence of phenolic compounds [57–59]. Park and John [60] identified eight phenolic acids in bamboo shoots of* Phyllostachys pubescence* of which protocatechuic acid, p-hydroxybenzoic acid, and syringic acid were found to be most abundant. The extracts of stems and leaves of* Phyllostachys *spp. exhibited strong antibacterial activities [61]. Velioglu et al. [62] reported that the correlation between the antioxidant property of the plant and phenolic compound content was statistically significant. Zhang et al. [28] studied changes in amount of phenolic compounds when fresh bamboo shoots were boiled, steamed, and stir fried. The total phenolic content in both boiled and stirs fried samples reduced slightly compared to fresh shoots. But there was increase in the level of phenolic compounds in steamed bamboo shoot. The decrease in amount of phenolic compounds in boiled or stir fried shoot samples may be due to decomposition of phenolic compounds during the heat treatment. However, Yamaguchi et al. [63] opined that heat treatment may lead to the inactivation of existing phenolic oxidases which were responsible for decomposition of polyphenols. The higher level of total phenolic compounds and increase in antioxidant activity were positively correlated [64]. Pandey and Ojha [37] reported variation in phenolic compound present in bamboo shoots of* Dendrocalamus asper, Dendrocalamus strictus,* and* Bambusa tulda* at different optimum harvesting times. Gallic acid concentration increased in* Dendrocalamus asper* and so did the caffeic acid in all bamboo species with increase in age of the shoots. The level of vanillic acid in* Bambusa tulda* was decreased but its concentration increased in both* Dendrocalamus asper* (from 0.009 g/100 g fresh weight to 1.262 g/100 g fresh weight) and* Dendrocalamus strictus* (from 0.273 g/100 g fresh weight to 2.563 g/100 g fresh weight). Pandey and Ojha [32] also observed decrease in total phenol content in shoots of* Bambusa bamboos, Bambusa tulda,* and* Dendrocalamus asper* when shoots were boiled in higher salt concentration for longer duration.

## 3. Taxiphyllin in Bamboo Shoots

**Figure 1:**
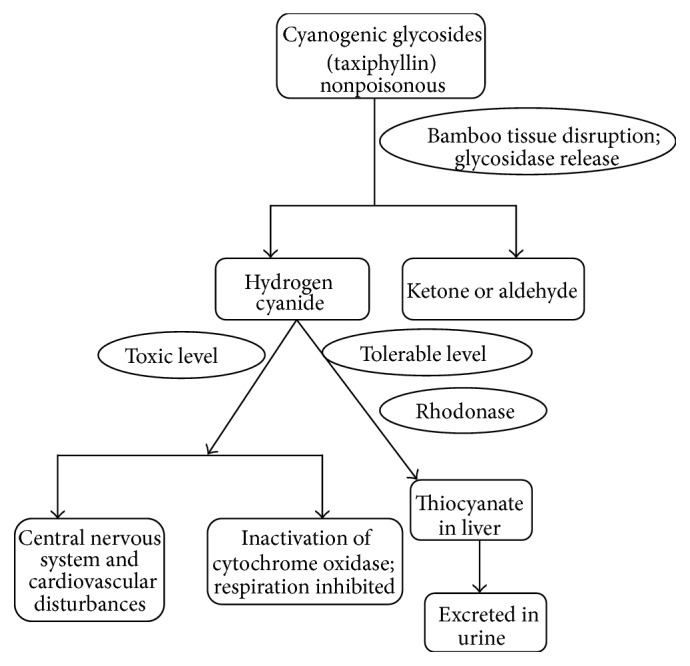
Hydrogen cyanide production in bamboo shoot and its effect on human health.

Bamboo shoots contain varying amount of cyanogen glycosides called taxiphyllin [65–68]. The *β*-glycosidase which is released in disrupted bamboo shoot tissues acts on taxiphyllin to produce harmful hydrogen cyanide whose level should not exceed the toxic level in humans [69]. The harmful hydrogen cyanide should be removed if shoots are to be used for human consumption [70]. The generation of cyanide in bamboo shoots and its possible harmful effects on human health in its toxic level are represented in Figure 1. Many investigators have worked on different bamboo species to determine the cyanogen contents [65, 67, 71]. Jones [72] reported variation in cyanide (HCN) content in different parts of the bamboo plant and also with different species of bamboos. Bamboo shoots of most edible species contain high amount of cyanogen glycoside with its maximum concentration found at the shoot tip. Haque and Bradbury [73] observed total cyanide content in bamboo shoots of* Bambusa arundinacea* (1010 ppm) to be much higher than other plants like flax seed meal (390 ppm), cassava root (27 ppm), sorghum leaf (750 ppm), apricot stone (785 ppm), and giant taro leaf (29 ppm). Unlike linamarin and lotaustralin which are the cyanogenic glycosides found in cassava plants, taxiphyllin in bamboo shoots is highly unstable and is easily decomposed when treated with boiling water. Bamboo shoot with high toxic level of cyanogenic compound can be made totally edible when detoxify by boiling in water for around 2 hours. However, consumption of improperly prepared or processed bamboo shoots may produce symptoms like rapid respiration, drop in blood pressure, dizziness, stomach pains, headache, vomiting convulsion, and coma [74]. Hydrogen cyanide interferes in proper functioning of cytochrome oxidase inhibiting normal cellular respiration. According to FSANZ report, the HCN present in concentration of 0.5–3.5 mg/Kg body weight is considered as lethal which may cause serious health damage leading to death. HCN is a respiratory poison which when present in high concentration may lead to serious health predicament to individual concerned. Pramod Kumar et al. [75] reported a case of cyanide poisoning in JSS Hospital, Mysore, India, when a 14-year-old female patient was brought to the Hospital in unconscious state with a history of consumption of bamboo shoot extract (juice). The patient showed the symptoms of convulsion, vomiting, respiratory distress, and loss of consciousness but regained consciousness two days after proper medical treatment. This case might be an isolated one as there have not been any reports of extreme cyanide poisoning because of bamboo shoot consumption as fresh or fermented shoots are presoaked and boiled in water during the preparation of most bamboo based ethnic dishes. Some tribal people in Northeast India consume aqueous exudates of fermented bamboo shoots apart from normal fresh and fermented shoots as one of the important condiments providing ethnic flavour to various traditional dishes. Incidence of stomach ailments and other mild health related problems are evident when improperly processed bamboo shoots are consumed. Shoot processing methods presently employed by locals are largely crude and traditional and lack scientific approaches for generation of safe bamboo foods. Devising scientific processing methods to reduce cyanogenic content and also assessing the toxicity of processed bamboo foods are essential to avoid acute cyanide poisoning during consumption. Various methods are being adopted to lower cyanide content and remove bitterness from either fresh or fermented shoots for safe and healthy consumption. Cooking at around 100°C for 48 hours effectively eliminates 97% of cyanide content of the shoot [76]. Other simple procedures include slicing of tender shoots into thin slices, drying, and boiling in salt water followed by draining. The fresh shoots must be adequately processed before consumption to remove the toxic and bitter components [77, 78] that showed reduction of cyanide content in fresh bamboo shoot when they were subjected to NaCl treatment. The hydrocyanic acid content of bamboo shoot can be significantly lowered during the time of bamboo shoot steaming process [79]. Pandey and Ojha [32] observed that bamboo shoots processed by boiling in water with varied concentration of NaCl for different time intervals produced maximal cyanide removal with minimum nutrient loss. The cyanogen in shoots of* Bambusa tulda* and* Dendrocalamus asper* was significantly lowered without much reduction in the content of carbohydrates, proteins, total phenol, potassium, phosphorus, and sodium when shoots were boiled for 25 minutes in 1%, 5%, and 10% NaCl solution. Wongsakpairod [80] showed that HCN can be removed by exposing bamboo shoots to superheated steam as taxiphyllin decomposed at over 116°C. Bhardwaj et al. [81] also reported the use of banana leaves by* Adi* tribe of Arunachal Pradesh for removing toxic cyanogen during bamboo fermentation. The bamboo shoots were pressed hard under big stones and kept for 3-4 months to lower down the bitter taste of fermented bamboo shoots. Choudhury et al. [82] identified the importance of devising proper shoot processing methods to generate healthy improved bamboo shoot product with longer shelf life.

## 4. Different Traditional Edible Forms of Bamboo Shoot

The emerging tender shoots must be collected at appropriate time of growing season when young shoots attend suitable height and development. The best edible shoots for consumption are obtained in middle of growing season as shoots harvested during the beginning or end of growing season tend to be either weaker or overdeveloped. The bamboo shoots may be further processed for consumption in fresh or fermented forms after harvest. They are popular food items and are used to prepare different traditional dishes in many Asian countries like China, Korea, Japan, Thailand and Taiwan.* Gulai rebung* which is made from bamboo shoots with thick coconut milk is widely consumed in Indonesia. People of Philippines make local cuisine called* labong* from bamboo shoots, coconut milk, and chilies [83]. A popular local dish called* Alui tama* is consumed in Nepal which is made by cooking the fermented bamboo shoots with potatoes. The young fresh bamboo shoots which are called* tusa* in hills of Nepal and fermented ones identified as* tama* are considered as important ethnic food items of the region [84]. The steamed ground pork patty with finely diced bamboo shoots sprinkled with soy sauce on the top is consumed as one of the most popular Chinese dishes. The freshly harvested bamboo shoots after the removal of hard sheaths are cleaned with water and shredded in small slices. The shoot pieces are treated with plain water for 2 to 3 hours or boiled for 1 and half hours to remove the acridity. In some countries like Thailand and Vietnam, the finely chopped bamboos may be directly consumed as salad after water treatment. The bamboo shoot pieces after boiling are salted slightly for 8–10 minutes and consumed in Australia and New Zealand [85]. Popular Thai curry called* ma khua proh* is made by cooking bamboo shoots with white pumpkin and pea aubergine.* Dom jud nomai* is another highly sought after Thai soup made from thinly sliced bamboo shoots with pork ribs mixed with salt, garlic, and pepper. The Vietnamese broth called* Súp bún mang gá *which is a noodle soup made with chicken and fresh bamboo shoots is usually taken as breakfast. The freshly collected bamboo shoots can be canned after proper processing. China, Taiwan, and Thailand are the leading countries in the export of canned bamboos to other countries like USA, UK, Australia, Singapore, Canada, and New Zealand [86]. There are reports of using bamboo shoots as food in other parts of India like Orissa, Gujarat, and Western Ghats regions of Karnataka [87]. The* Kandha* tribe of Western India prepares* kardi* a traditional bamboo based food by soacking shredded pieces of shoots in water for a day in order to remove bitterness before cooking [88]. Bal et al. [89] while undertaking a survey in five villages of Orissa, India, observed that bamboo shoots are consumed by locals after proper boiling in water. The processing of fresh shoots is performed by boiling at different temperature conditions and duration to remove the acrid taste and toxic elements. They are used in the preparation of pickles, snacks, papads, and other fried food stuffs in remote and hilly areas of Western Ghats [90].

## 5. Bamboo Based Traditional Foods of North-East India 

The Northeast India comprising of eight states, namely, Assam, Manipur, Meghalaya, Mizoram, Nagaland, Sikkim, Arunachal Pradesh, and Tripura is located between 21°–30°N latitude and 85°–98°E longitude. It covers an area of around 18.4 million hectares and is physiographical categorized into the Eastern Himalayas, Northeast hills, Brahmaputra, and Barak valley. This region with different ethnic communities and diverse cultures and religions is considered as a treasure house of bamboos contributing more than 66% of total bamboos resources available in India [91]. About 16 edible bamboo species are reported in Northeast India [92] and some of the important edible bamboo species are* Arundinaria callosa, Bambusa nutans, Bambusa pallida, Bambusa polymorpha, Bambusa tulda, Dendrocalamus hamiltonii, Dendrocalamus giganteus, Dendrocalamus brandisii, Melocanna baccifera, Melocanna bambusoides, Dendrocalamus hookeri, Dendrocalamus sikkimensis, Dendrocalamus strictus, *and* Phyllostachys manni. *The bamboo shoots form an integral part of many of popular traditional cuisines and the locals consumed either fresh or fermented bamboo shoots in the form of various local delicacies.

### 5.1. Fresh Bamboo Shoots

**Figure 2:**
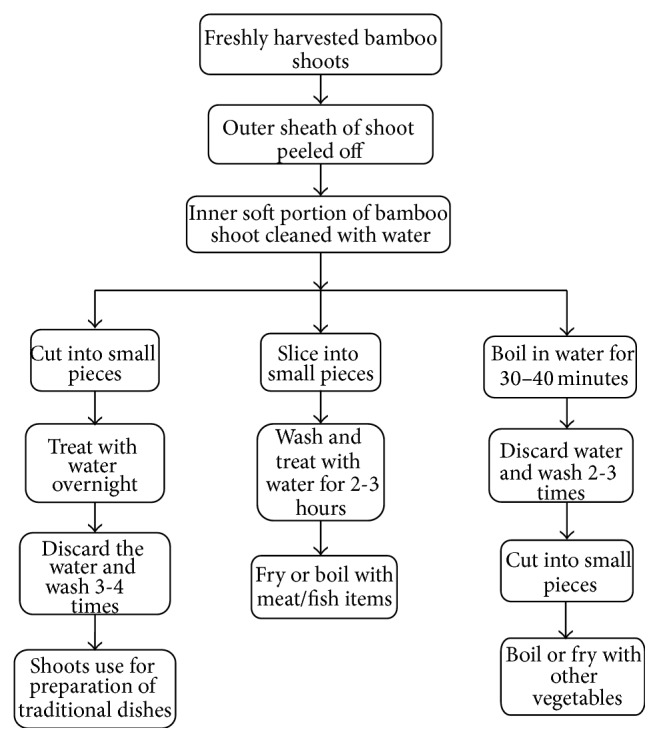
Use of fresh bamboo shoots for preparation of traditional foods.

**Figure 3:**
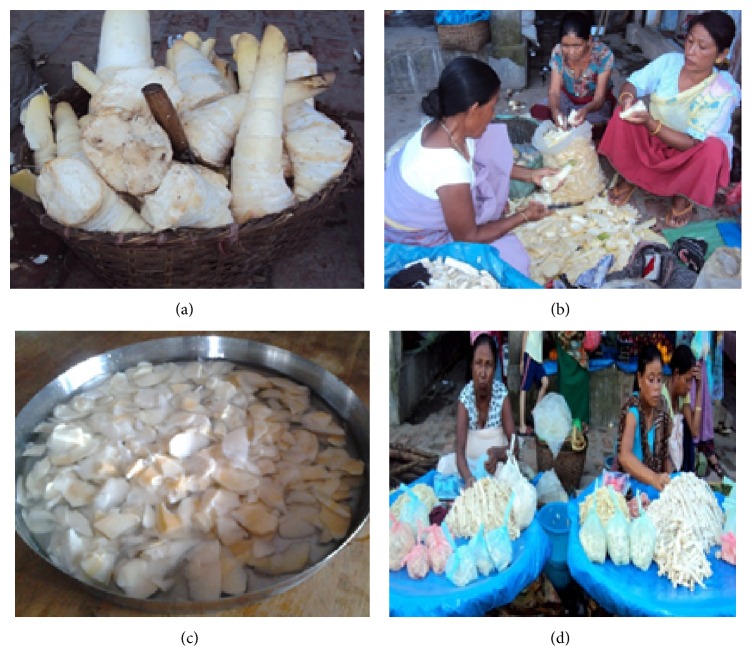
Production of traditional bamboo shoot foods. (a) The newly harvested bamboo shoots after removing the outer sheaths, (b) bamboo shoots chopped into fine pieces to make* ushoi*, (c) bamboo shoot pieces are treated in water before being utilised as food, and (d) fermented bamboo shoots sold in the market as vegetable.

The indigenous people of Northeast India take fresh shoots as one of the popular food items. The possible uses of fresh bamboo shoots for preparation of several traditional dishes are depicted in Figure 2. Fresh bamboo shoots are consumed in Manipur as* ushoi* which is produced by chopping the inner soft portion of shoot into thin slices after peeling off the outer sheath and treating them in water for 3-4 hours (Figures 3(a), 3(b), and 3(c)). It is primarily used in the preparation of number of favourite ethnic dishes and can be preserved for off-season by drying under sun after excess water is drained off. Another bamboo species popularly consumed fresh is* Arundinaria callosa* whose average size slender shoots with protective sheaths are sold in market during the harvesting season. The shoots after peeling off the hard sheaths are sliced into pieces which are either boiled for around 1 hour or shocked in water for 3-4 hours to remove bitterness of shoot. The properly processed shoots are used to make variety of traditional local chutney called* iromba* and also cook as vegetable with fishes and meat. In Arunachal Pradesh, young bamboo shoots are boiled and cut into pieces and used as vegetable for preparation of traditional dish called* kupe* [93]. Shoots obtained from* Bambusa balcooa*,* Bambusa nutans*, and* Dendrocalamus strictus* are slightly bitter and require proper processing by either steaming or boiling briefly and changing the water intermittingly till the bitterness is lost [7]. In states like Meghalaya, Mizoram, and Sikkim, the fresh young shoots of* Melocanna baccifera, Dendrocalamus hamiltonii, Bambusa balcooa, *and* Chimonobambusa hookeriana *are consumed either boiled with other leafy vegetables or fried with other nonvegetable components [94].

### 5.2. Fermented Bamboo Shoot

The bamboo shoots in fermented form are consumed as one of favourite traditional foods by different ethnic communities. Some of the important fermented bamboo shoot products familiar with locals are* soibum, soidon, soijim, bastanga pani, rep, eup, mesu, *and so forth*. Soibum* is one of the most popular fermented foods of Manipur normally available in almost all local vegetable markets (Figure 3(d)). It is made from tender shoots of* Dendrocalamus hamiltonii, Dendrocalamus giganteus, Bambusa tulda*,* Bambusa balcooa, *and* Bambusa pallid *and is widely used in preparation of special delicacies. Jeyaram et al. [95] reported two approaches of* soibum *production normally practised in the region. The more popular* kwatha* or* noney* method uses traditionally designed chamber lined with* Colocasia* leaves or polythene sheets and packed with thinly sliced bamboo shoots. While the* Andro* type involves the use of big earthen pot filled partly with shredded bamboo shoots, the quantity of which is increased by external addition as fermentation proceeds. The liquid portion is not removed as the fermentation continues for 6–8 months. A simpler method practised in* Bishenpur *is use of a large plastic tub which contains finely chopped shoots with enough water to submerge them; the shoots are kept in aerobic condition for 15–20 days before being removed and packed in closed plastic bags for 2-3 months. The liquid remnants can be repeatedly used as starter culture for next round of* soibum* production as it shortens the fermentation process by 6-7 days. Giri and Janmejay [25] indicated the increase in quality of* soibum *with increase in incubation period.* Tankhul *tribes use local bamboo variety called* ngathan* to produce dried* soibum *which looks like noodles with twisted appearances. In Arunachal Pradesh,* Nishi *tribes consume* ekung* a fermented bamboo shoot product similar to* soibum. Apatani *tribes of the same region prepare* hirring* by fermenting the sliced tender upper portion of shoots of* Dendrocalamus giganteus, Phyllostachys assamica *and* Bambusa tulda.* The delicious curries produced by cooking* hirring *together with meat, fish, or vegetables are favourites among locals [96].* Barman *community of Tripura prepares traditional dish called* godhak* which is made by mixing fermented bamboo shoots along with pseudobulbs of banana, dry fishes, salt, chili, and garlic [97].* Mesu *is another fermented shoot product which is a favourite among the people of Sikkim and Darjeeling as locally made pickle.* Naga* tribes of Nagaland use bamboo juice extracted from fermented shoots as integral component of daily cuisine as it adds ethnic flavour and aroma to the curries.

## 6. Market Potential of Bamboo Shoot in Northeast India

The value of international bamboo market is estimated to be around $10 billion [98]. The total revenue from bamboo shoots is about $1.2 billion while other bamboo based products accounts for around $3 billion [99]. Bamboo shoot is consumed worldwide and annual world bamboo consumption is presently over 2 million tonnes [100]. The intake of bamboo shoot as popular food is mostly found in Asian countries like China, Thailand, Korea, Taiwan, and Japan. Bamboo shoots occupy the second position in annual vegetable production volume in Taiwan with* Dendrocalamus latiflorus* and* Bambusa oldhamii* as the two most important edible bamboos consumed in the country. China and Thailand are considered as the two most dominant players in the international bamboo market. China is the largest exporter of edible shoot earning around $4 million annually with Thailand following closely at second position [101]. The trade value of bamboo shoots is found to be highest in all bamboo commodities being exported from China between 1998 and 2002. China exports bamboo shoots mainly to Japan and USA accounting approximately about 74% and 11%, respectively, of total bamboo exports. Japan imports 134000 tonnes of bamboo shoots per annum amounting to 45% of world total import, while USA stands distant second by importing around 44000 tonnes annually [82]. The import of bamboo shoots in Australia in 1992 was estimated at 1,350 tonnes accounting to about $5.0 million value [102]. India has rich bamboo genetic resources and is the second largest bamboo producing nation in the world next to China with annual production of 32.3 million tonnes [103, 104]. However, domestic bamboo shoot industry of the country is at nascent stage which is estimated at around $0.8 million. The general acceptability of bamboo shoot as popular food among the masses is low due to its pungent taste and strong odour as well as the easy availability of lower priced vegetables as an alternative. As largely bamboo shoots are consumed traditionally by ethnic people, the popularity of the food is confined to only few tribes and communities and the taste has to be developed through frequent exposure. In India, bamboo shoots are consumed mainly in Northeast India where they are integral part of many popular tribal cuisines. The prospect of bamboo shoot based industry especially in the region is tremendous as it harbours more than 66% of total bamboo population of India. Despite being a rich repository of bamboos, this region produces only 5685 tonnes of bamboo shoots annually [9]. Singh [105] reported a gross annual income of $1.3 million in 8 Northeastern states of India in bamboo shoot trade which included marketing of fresh and processed bamboo shoots. The net annual income from bamboo shoot industry is $0.7 million indicating one of the potentially profitable business avenues for the region. However, the present market scenario of bamboo shoot is not very promising despite its very rich bamboo resources. The production of bamboo shoot is mainly for domestic and local consumptions and it has not been pursued at the industrial scale. There are very few canning factories which can totally absorb production of perishable bamboo shoot products. There are only 3-4 small processing units in entire Northeast region each located at Dimapur, Nagaland (900 tonnes/year), Jorhat (200 tonnes/year), and Bongaigoan, Assam (300 tonnes/year). The volume of bamboo shoot export outside Northeast region is very less and majority of locally produced bamboo shoot products find their way to local markets only. In order to tap the highly potential market, more efforts need to be made with outmost sincerity to exploit the rich bamboo resource by encouraging local bamboo shoot production at the industrial scale using proper scientific methods. Nowadays with increased consciousness for safe and healthy foods and the people's preference for bamboo based Chinese and Oriental cuisines, the prospect of bamboo shoot in local and international market is tremendous. Moreover, demand for bamboo shoot has increased several folds as many speciality restaurants, food outlets, and 5 star hotels in towns and metropolitan cities of India serve bamboo based continental and oriental foods. The domestic consumption of bamboo shoot in Japan, Korea, and other Asian countries is also increasing and the demand for import is becoming more as the bamboo shoot production in these countries has declined due to short production season. The market potential of bamboo in India is estimated to be around $6 million by 2015 and the Northeast region being the largest contributor of bamboos in India, there is an ample opportunity to step up its own bamboo shoot production to not only address its own local requirement but also exploit the export avenues especially the highly potential and profitable destinations like Japan, Korea, Taiwan, and other Asiatic countries.

## 7. Bamboo Micropropagation: A Biotechnological Approach to Effective Conservation 

The bamboos are largely overexploited because of their enormous utility for different purposes besides being used as popular ethnic food [106]. There is alarming reduction of bamboo natural resources due to their increase utilization for industrial and domestic purposes and also widespread habit destruction [107]. Propagation of bamboos through conventional methods by using seeds, rhizome division, and culm cutting do not produce desired result because of nonavailability of adequate planting materials, problems in handling of large size propagules, unpredicted flowering cycle, and short viability of the seeds [108]. As bamboo propagation through classical methods cannot generate enough bamboos resources, there is a necessity of adopting new methods to increase bamboo production at much larger scale [109]. Micropropagation of bamboos through tissue culture provides an excellent opportunity as an alternative biotechnological tool to insufficient and inefficient classical propagation methods for rapidly propagating several rare and edible bamboos in large numbers [110]. Several workers have successfully micropropagated bamboos using different explants like mature embryos, rhizomes, nodal segments, and inflorescences [111–117]. The selection of appropriate explants for culture initiation and further regeneration is paramount to success of bamboo tissue culture. The collection of explant in winter produced less contamination as compared to explants collected during rainy season. The surface disinfection of selected bamboo explants can be effectively performed using either mercuric chloride (0.1–1%) or sodium hypochlorite (1-2%) along with treatment of soft detergent solution [118]. Mishra et al. [115] administered 0.05% mercuric chloride for nodal explants collected during winter time while 0.1% mercuric chloride treatment was given to explants harvested during summer season. The use of 0.1-0.2% mercuric chloride in rainy season enhanced aseptic culture establishment but inhibited bud break due to its toxicity to explant tissues. Fungicides (bivastin 1%) and bacteriomycin (streptomycin 0.2%) may also be employed if required to control fungal and bacterial contaminations of selected nodal explants. Bud break and sprouting of buds from nodal explant is strongly influenced by genotype, juvenility of explant tissues, and its physiological state, season of explant collection, and culture initiation [119–121]. Sharma and Sarma [122] reported axillary bud breaking 3 weeks in* Bambusa balcooa *when nodal explants were transferred on BAP (6-benzylaminopurine) and IBA (indole-3-butyric acid) supplemented agar solidified Murashige and Skoog [123] medium. However, sprouting of axillary was also noticed in 4 weeks on phytagel solidified MS medium without any growth regulators [124]. The bud break and bud sprouting were recorded in just 6-7 days when the nodal explants of* M. baccifera* were inoculated in liquid MS medium enriched with 14 mg/L Kn (kinetin) and 2 mg/L BAP. The bud break and sprouting took more time in solid medium with same hormonal combinations. The improved frequency of bud break and shoot multiplication and elongation were also observed by several workers [113, 125, 126]. The lower growth response of culture in agar gelled nutrient medium may be due to binding of water, absorption of nutrients, and plant growth regulators (PGRs) by solubilised agar which led to reduce utilization of nutrients, PGRs, and other essential constituents by the culture. The frequency of bud break and incidence of bacterial contamination are dependent on season for explants collection. The bud break was found to be low for explants collected during rainy reason due to high bacterial and fungal contamination [127]. The shoot elongation and proliferation after the bud break could be increased and maintained by subculturing at regular interval of 4-5 weeks on MS medium incorporated with different concentrations of growth regulators [119]. Sharma and Sarma [122] observed stagnant and unhealthy growth of shoot after bud break when subculturing was performed at lower concentration of Kn in* Bambusa balcooa*. The stunted growth if continues for longer time will ultimately lead to death of culture. Regular subculturing provides optimal and desired level of culture regeneration and multiplication and delay in doing so will produce death of culture due to browning. The phenolic compounds responsible for browning phenomenon in culture can be controlled by frequent subculturing and incorporating ascorbic acid, activated charcoal, or polyvinyl pyrrolidone. Singh et al. [128] reported significant reduction of browning by supplementing medium with ascorbic acid, while activated charcoal and polyvinyl pyrrolidone did not have any effect on the control of phenolic compounds. Shoot multiplication and elongation were more pronounced with well-developed leaves in* M. baccifera* and* D. hamiltonii* when subculturing was done every 15–20 days in MS medium supplemented either with 0.5 mg/L NAA (1-naphthaleneacetic acid) and 5 mg/L Kn or 2 mg/L Kn, 0.5 mg/L IBA, and 0.3% activated charcoal [129]. Similar observation was reported by Mudoi and Borthakur [130] in* Bambusa balcooa. *Prominent shooting response was detected in* Bambusa bamboos* when elongated axillary shoots from primary culture were transferred to shooting medium incorporated with different concentrations of BAP [131]. The main constraint to successful propagation of bamboos and other woody species is the difficulty in reducing appropriate rooting [132, 133]. The use of auxins such as IBA and NAA was reported for effective rooting induction in several bamboo species [134–136]. However, rooting in* Pleioblastus pygmaeus* was successfully established in basal MS medium without any growth regulators [137]. Incorporation of 5.1 mg/L IBA and 0.01 mg/L BAP in liquid MS medium produced maximum rooting response in* Arundinaria callosa* Munro [138]. Stout rooting system was observed in 7–10 days when the* in vitro* rooted shoots were transferred to PGR-free half strength MS medium. Use of additives like choline chloride and coumarine along with auxins enhanced rooting induction in some bamboos [120, 129]. Two steps rooting procedure in bamboos was formulated by Bag et al. [139] when they used IBA treatment for rooting induction which is followed by transferring the culture to auxin-free media to achieve strong rooting system. The regenerated plantlets with established shoots and roots were washed in running tap water and shifted to soil mixture in a pot for appropriate hardening. The well-rooted plants after proper hardening have survival rate of about 80–85% under polyhouse/net house conditions which can be transplanted to field for effecting ecorestoration in their natural habitats. The use of tissue culture technology can propel the production of bamboos at the scale of more than 500000 bamboo plants per year [140]. This scientific approach of rapid and mass scale propagation of bamboos can effectively restore the already depleted natural bamboo resources. The “*ex situ*” approach of bamboo conservation may also be adopted by declaring the bamboo dominated forest areas as special gene sanctuary for bamboos. Establishment of “Bambusetum” at suitable places is another approach to effective bamboo conservation at smaller scale. The conservation of bamboos at Cherra and Basa in Arunachal Pradesh is an appropriate example where 35 species and 30 species of bamboos are, respectively, being preserved successfully [141]. The* in vitro* and* ex situ* conservation methods if effectively formulated and implemented may help address to a large extent the present acute scenario of population depletion of bamboo species of the region.

## 8. Conclusions and Future Perspective

Bamboo shoots have immense potential of being used as important health food as they have high content of useful proteins, amino acids, carbohydrates, and many important minerals and vitamins and very low fat. Bamboo shoots are consumed predominantly in Asiatic countries where they form integral part of several traditional cuisines of the region. The usefulness of bamboo shoots as health food is not largely known by general public due to ignorance of their high nutritional values. There is a greater necessity to create awareness among the people about their nutritional health benefits so that they are widely accepted. Bamboos occupy a very significant position in everyday life of indigenous people of Northeast India due to their enormous utility as traditional food, house construction materials, and raw materials for production of useful domestic and other handicraft items. The fresh or fermented bamboo shoots form an indispensable part of number of ethnic dishes. But shoots should be properly processed as they contain high level of toxic cyanogenic glycosides. Using improved shoot processing methods based on scientific approach instead of crude and unhygienic ones will not only reduce toxic cyanogenic compound but also retain the nutritional values. The region being the largest producer of bamboos in India has a bright prospect for bamboo shoot industry but presently bamboo shoot production is predominantly for fulfilling the local needs. There is a need to increase bamboo processing and packaging units as very few operate actively in the region. Effective marketing strategies should also be formulated to extract maximum profits by making bamboo shoot products available to as many potential and prospective customers inside and outside the country. Intervention of modern micropropagation techniques is also essential to control the falling population of bamboos. The local bamboo shoot industry if properly established will not only help in socioeconomic upliftment of the region but also generate huge income for the country.

## Figures and Tables

**Table 1:** Comparison of nutrient composition of young bamboo shoots of *Bambusa tulda* and *Dendrocalamus hamiltonii* with some common vegetables [7].

Nutrients	*Bambusa tulda *	*Dendrocalamus hamiltonii *	*Daucus carota *	*Raphanus sativus *	*Spinacea oleracea *	*Solanum tuberosum *	*Abelmoschus* *esculentus *	*Curcuma sativus *	*Cucurbita* *maxima *
Amino acids (g/100 g)	3.65	3.18	0.20	0.40	0.30	0.20	0.30	0.10	0.20
Protein (g/100 g)	3.69	3.72	0.90	0.70	2.00	1.60	1.90	0.60	1.40
Carbohydrate (g/100 g)	6.92	5.50	10.60	3.40	2.90	22.6	6.40	2.50	6.50
Fats (g/100 g)	0.48	0.41	0.10	0.40	0.70	0.10	0.20	0.10	0.40
Fibers (g/100 g)	3.97	3.90	1.20	0.60	2.00	0.40	1.20	0.40	1.10
Vitamin C (mg/100 g)	1.42	2.45	15.00	1.60	0.60	0.40	1.20	0.70	0.07
Vitamin E (mg/100 g)	0.61	0.71	3.00	15.00	28.10	19.70	13.00	3.20	0.90
Potassium (mg/100 g)	408	416	108.00	393.00	558.00	424.00	103.00	135.00	340.00
Iron (mg/100 g)	3.19	2.69	1.03	1.00	2.70	0.80	0.35	0.90	0.80
Calcium (mg/100 g)	4.06	3.00	80.00	35.00	9.90	12.00	56.00	14.00	21.00
Cupper (mg/100 g)	0.44	0.29	0.10	0.02	0.10	0.16	0.11	0.09	0.10
Sodium (mg/100 g)	19.96	9.32	35.60	39.00	79.00	11.00	6.90	2.00	5.60
Zinc (mg/100 g)	0.72	0.70	0.36	0.30	0.50	0.30	0.42	0.23	0.30

**Table 2:** Nutrient content of freshly harvested shoots of some bamboo species [7–9].

Nutrients	*B*. *bamboos *	*B*. *nutans *	*B*. *polymorpha *	*B*. *tulda *	*B*. *vulgaris *	*D*. *asper *	*D*. *giganteus *	*D*. *strictus *
Amino acids (g/100 g)	3.98	3.89	3.42	3.65	3.57	3.12	3.96	3.07
Protein (g/100 g)	3.57	2.84	3.64	3.69	3.64	3.59	3.11	2.60
Carbohydrate (g/100 g)	5.42	5.47	5.44	6.92	6.51	4.90	5.10	6.17
Fats (g/100 g)	0.50	0.40	0.46	0.48	0.50	0.40	0.39	0.33
Fibres (g/100 g)	4.49	2.28	3.81	3.97	4.24	3.54	2.60	2.26
Vitamin C (mg/100 g)	1.90	1.19	2.60	1.42	4.80	3.20	3.28	2.43
Vitamin E (mg/100 g)	0.61	0.47	0.49	0.61	0.52	0.91	0.69	0.58
Calcium (mg/100 g)	0.36	1500	180.69	1300	320	5.51	26.93	139.5
Phosphorus (mg/100 g)	30.12	900	15.06	700	220	40.95	12.57	58.13
Iron (mg/100 g)	3.00	-	1.53	1.57	-	3.37	1.06	2.91
Sodium (mg/100 g)	10.10	-	-	12.96	400	10.14	3.64	0.08
Potassium (mg/100 gm)	-	30.0	-	20.0	920	464	275	-
Magnesium (mg/100 gm)	5.38	40.0	-	40.0	100	10.14	9.57	0.17

Note: Hyphen (-) in table indicates authentic data not available for the particular bamboo species.

**Table 3:** Nutrient composition of raw, boiled, and fermented shoots of different bamboo species [6, 8, 10, 11].

Nature of shoots	Bamboo species	Carbohydrate (g/100 g)	Protein (g/100 g)	Fats (g/100 g)	Vitamin C (mg/100 g)	Vitamin E (mg/100 g)	Fibres (g/100 g)
Raw shoots	*Dendrocalamus strictus *	85.98	21.51	0.10	5.80	-	0.98
	*Dendrocalamusasper *	2.90	25.80	3.59	3.30	0.91	0.70
	*D. giganteus *	5.10	-	0.39	3.28	0.69	-
	*Bambusa vulgaris *	3.40	25.70	0.20	13.70	-	0.70
	*Bambusa nutans *	3.30	21.10	1.00	5.30	-	0.76
Boiled shoots	*Dendrocalamus strictus *	5.00	-	-	-	-	0.96
	*Dendrocalamus asper *	3.10	17.10	-	-	-	0.70
	*D. giganteus *	-	11.66	-	-	-	-
	*Bambusa vulgaris *	5.00	13.50	-	-	-	0.96
	*Bambusa nutans *	5.10	17.30	-	-	-	0.75
Fermented shoots	*Dendrocalamus strictus *	-	-	-	-	-	-
	*Dendrocalamus asper *	-	-	-	-	-	-
	*D. giganteus *	-	-	-	0.32	1.09	0.21
	*Bambusa vulagaris *	-	-	-	-	-	-
	*Bambusa nutans *	-	-	-	-	-	-

Note: Hyphen (-) in table indicates authentic data not available for the particular bamboo species.
